# *dnd*DB: A Database Focused on Phosphorothioation of the DNA Backbone

**DOI:** 10.1371/journal.pone.0005132

**Published:** 2009-04-09

**Authors:** Hong-Yu Ou, Xinyi He, Yucheng Shao, Cui Tai, Kumar Rajakumar, Zixin Deng

**Affiliations:** 1 Laboratory of Microbial Metabolism and School of Life Sciences & Biotechnology, Shanghai Jiaotong University, Shanghai, People's Republic of China; 2 Department of Infection, Immunity and Inflammation, Leicester Medical School, University of Leicester, Leicester, United Kingdom; 3 Department of Clinical Microbiology, University Hospitals of Leicester NHS Trust, Leicester, United Kingdom; University of British Columbia, Canada

## Abstract

**Background:**

The Dnd DNA degradation phenotype was first observed during electrophoresis of genomic DNA from *Streptomyces lividans* more than 20 years ago. It was subsequently shown to be governed by the five-gene *dnd* cluster. Similar gene clusters have now been found to be widespread among many other distantly related bacteria. Recently the *dnd* cluster was shown to mediate the incorporation of sulphur into the DNA backbone via a sequence-selective, stereo-specific phosphorothioate modification in *Escherichia coli* B7A. Intriguingly, to date all identified *dnd* clusters lie within mobile genetic elements, the vast majority in laterally transferred genomic islands.

**Methodology:**

We organized available data from experimental and bioinformatics analyses about the DNA phosphorothioation phenomenon and associated documentation as a *dnd*DB database. It contains the following detailed information: (i) Dnd phenotype; (ii) *dnd* gene clusters; (iii) genomic islands harbouring *dnd* genes; (iv) Dnd proteins and conserved domains. As of 25 December 2008, *dnd*DB contained data corresponding to 24 bacterial species exhibiting the Dnd phenotype reported in the scientific literature. In addition, via *in silico* analysis, *dnd*DB identified 26 syntenic *dnd* clusters from 25 species of Eubacteria and Archaea, 25 *dnd*-bearing genomic islands and one *dnd* plasmid containing 114 *dnd* genes. A further 397 other genes coding for proteins with varying levels of similarity to Dnd proteins were also included in *dnd*DB. A broad range of similarity search, sequence alignment and phylogenetic tools are readily accessible to allow for to individualized directions of research focused on *dnd* genes.

**Conclusion:**

*dnd*DB can facilitate efficient investigation of a wide range of aspects relating to *dnd* DNA modification and other island-encoded functions in host organisms. *dnd*DB version 1.0 is freely available at http://mml.sjtu.edu.cn/dndDB/.

## Introduction

The Dnd DNA degradation phenotype was observed during normal and pulsed-field gel electrophoresis of genomic DNA from *Streptomyces lividans* strain 66 [1]. DNA degradation during electrophoresis in the presence of tris, a commonly used biological buffer, has also been reported in many other distantly related bacterial species, such as *Escherichia coli, Salmonella enterica, Klebsiella pneumoniae, Vibrio parahaemolyticus, Pseudomonas aeruginosa, Pseudomonas fluorescens, Mycobacterium abscessus, Clostridium botulinum, and Clostridium difficile*. The Dnd phenotye was thought to involve a post-replicative DNA modification that rendered DNA susceptible to degradation at the electrophoretic anode. In 2005, the five-gene *dndABCDE* cluster responsible for this phenotype was identified in *S. lividans* [2]. Zhou *at al*. [2] demonstrated that the affected DNA had been modified *in vivo* by the addition of a sulphur-containing molecule through a likely biochemical pathway mediated by enzymes encoded by the *dnd* locus.

More recently the *dnd* cluster was shown to mediate the incorporation of sulphur into the DNA backbone via a sequence-selective, stereo-specific phosphorothioate modification in *E. coli* B7A [3]. By using high-performance liquid chromatography and mass spectrometry, the chemical structure of phosphorothioated DNA was determined revealing a sulfur atom in place of one of the nonbridging oxygen atoms on a DNA backbone-borne phosphate group. To our knowledge, this was the first report of natural modification of the DNA backbone itself and sets it apart from well-documented DNA methylation and other changes to DNA bases.

Intriguingly, the *S. lividans dnd* cluster lay within a large, mosaic genomic island named SLG [4], [5]. To date all 26 identified *dnd* clusters are borne on likely mobile genetic elements, twenty-five of which are harboured on genomic islands, fragments of alien DNA that have been incorporated into chromosomes of new hosts via horizontal gene transfer events [6].

The observed Dnd phenotype and recent microbiological, genetic and biochemical advances in the field have been reported in the scientific literature. However, disparate PubMed references and individual genome annotation and protein data deposited in public databases do not provide a unified resource required to facilitate the advanced searches, analyses and data manipulation necessary to fully exploit the available and rapidly emerging new data in the Dnd field. Consequently, we have created a MySQL database, *dnd*DB, to efficiently organize all available data from experimental and bioinformatics analyses about the phosphorothioation of DNA in Eubacteria and Archaea and provide a central repository of associated documentation. We propose that our evolving, web-based *dnd*DB resource will stimulate and facilitate research into many key questions, including the mechanism of sulfur incorporation, the biological significance of this DNA modification, the role, source and mode of dissemination of *dnd*-bearing genomic islands, and the potential for exploitation of these systems for biotechnological applications.

## Results and Discussion

The purpose of *dnd*DB is to provide a user-friendly interactive platform not only to efficiently archive, analyse and manipulate increasing data about bacterial and archeal *dnd* genes, linked island-borne genes, matching sets of cognate proteins, and the DNA phosphorothioation process itself, but to also empower researchers from different backgrounds to explore novel angels potentially related to this, thus far, unique DNA backbone modification process. A broad range of similarity search, sequence alignment and phylogenetic tools are readily accessible to allow for user-directed interrogation of the database, examination of user-supplied sequences and other individualized directions of research.

### User interface

The *dnd*DB homepage contains the following interfaces: ‘Introduction’ (Dnd background and references), ‘Dnd phenotype’ (experimental protocol and archived literature), ‘Gene&Cluster’ (degenerate primers, gene homologues and clusters), ‘Genomic island’ (genomic context), ‘Protein&Domain’ (putative function, homologues, conserved domains and references), ‘Search’ (search Dnd phenotype, gene or protein homologues by organism name), ‘Blast vs *dnd*DB’ (gene/protein sequence BLAST against *dnd*DB), ‘tBlastn for Dnd’ (Dnd protein prediction in user-supplied nucleotide sequence), ‘Restrict_Modifica’ (Dnd-dependent restriction-modification system), ‘Chemistry’ (sequence- and stereo-specific nature of DNA phosphorothioation), ‘Potential Applications’, ‘Useful Links’ and ‘Contact Us’.

### Organisms exhibiting the Dnd phenotype

**Figure 1:**
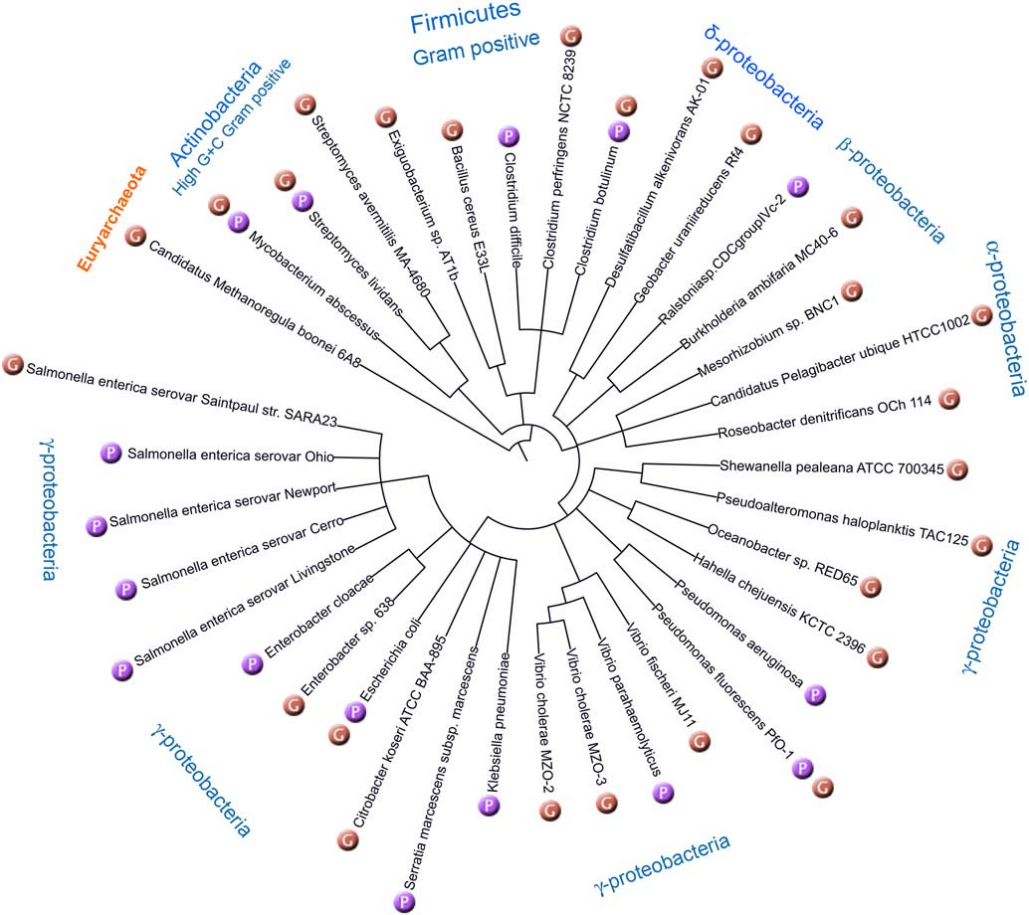
Inferred phylogenetic relationship of the 31 bacterial and one archael organism carrying known *dnd* clusters (denoted by orange ‘G’ balls) and/or documented to exhibit the Dnd phenotype (denoted by purple ‘P’ balls). The tree shown was constructed on the basis of NCBI taxonomy (http://www.ncbi.nlm.nih.gov/Taxonomy/) by using iTOL [11], which is now accessible via *dnd*DB.

Electrophoresis-associated DNA degradation, otherwise known as the Dnd phenotype, is a puzzling and long-standing phenomenon frequently observed during pulsed field gel electrophoresis (PFGE), when instead of discrete bands a smear pattern results. The current version of *dnd*DB includes a description of the Dnd phenotype in 24 bacterial species based on information extracted from PubMed references. The phylogenetic diversity and wide prokaryotic representation of these Dnd phenotype-positive organisms and others that we have shown to harbour *dnd* gene clusters is shown in Figure 1. These data are tabulated and easily retrieved using the ‘Search’ tool in *dnd*DB. In addition, users can download an optimized Dnd phenotype verification protocol which utilizes activated tris-acetate-EDTA (TAE) buffer during agarose gel electrophoresis to check the Dnd phenotype of bacterial strains of interest. A simple PCR-based protocol to identify potential *dndC* gene homologues in bacterial isolates developed using *dnd*DB is also provided. This method is also intended to serve as a template for other *dnd*DB-facilitated PCR-based screening assays.

### Horizontal gene transfer

Comparative analysis of *dnd* genes at a variety of granularities, such as the single gene, gene cluster, genomic island or genome-scale level, will greatly aid investigations into the evolution of *dnd* gene clusters and the mechanisms that brought about their widespread dissemination across diverse and distant bacterial species. In *dnd*DB, a powerful multiple sequence alignment algorithm, Muscle v3.7 [7], and a Java alignment editor, JalView 2.4 [8], were integrated to facilitate the comparison of the *dnd* gene clusters from 24 taxonomically distinct bacterial species and one archael member from various geographic niches. In addition, the popular GBrowse viewer [9] that combines a database and interactive web page was employed for manipulating and displaying annotations on *dnd*-bearing genomes. Remarkably, all identified *dnd* clusters lay within larger mobile genetic elements, 23 within chromosomal islands, 2 in the islands in the plasmid-derived chromosome II of *Pseudoalteromonas haloplanktis* TAC125 and *Vibrio fischeri* MJ11, and one on the large Plasmid 3 of *Mesorhizobium* sp. BNC1 (see Table 1 for details). Analysis of these putative *dnd*-encoding islands demonstrated common key features typical of GIs: organism-atypical G+C contents, integration into tRNA genes, and/or possession of terminal direct repeats, integrase- and/or transposase-encoding sequences. Phylogenetic analysis of the *dnd* genes in the 26 identified *dnd* clusters confirmed the diverse nature of these sequences. Furthermore, significant discordances between the 16S rDNA- and *dnd*-derived phylogenetic trees, marked differences in the gene content within the remainder of the *dnd i*slands, and the frequent absence of *dnd* islands in members of the same species, strongly supported the notion that the diverse *dnd* clusters and their cognate islands had been acquired independently on many occasions, rather than arising from a single or limited number of vertical evolutionary events. However, to date none of the defined *dnd* islands have been shown to be functionally mobile, though at least one, the *S. lividans* SLG island, is known to function as a typical, self-circularizing, site-specific integrative element [5].

**Table 1:** *dnd* clusters present on mobile genetic elements comprising 25 genomic islands and one plasmid

Organism [NCBI RefSeq accession no.]	*dnd* gene order a	GI size (kb)	GI content (%) [genome G+C content]	No. of integrase/transposase genes
*Bacillus cereus* E33L [NC_006274]	*ABCDE*	33.7	32.6% [35.4%]	0
*Burkholderia ambifaria* MC40-6 [NC_010551]	*BCDE*	12.4	56.4% [66.0%]	0
*Candidatus Methanoregula boonei* 6A8 [NC_009712]	*ABCDE*	11.3	40.2% [54.5%]	0
*Candidatus Pelagibacter ubique* HTCC1002 [NZ_AAPV01000002]	*ABCDE*	38.0	28.0% [29.8%]	0
*Citrobacter koseri* ATCC BAA-895 [NC_009792]	*BCDE*	46.6	40.0% [53.8%]	0
*Clostridium botulinum* E3 str. Alaska E43 [NC_010723]	*BCDE*	20.7	25.9% [27.4%]	0
*Clostridium perfringens* NCTC 8239 [NZ_ABDY01000007]	*BCDE*	18.3	25.5% [28.7%]	0
*Desulfatibacillum alkenivorans* AK-01 [NZ_ABII01000002]	*BCDE*	28.8	42.3% [54.5%]	1
*Enterobacter* sp. 638 [NC_009436]	*BCDE*	16.9	44.3% [53.0%]	1
*Escherichia coli* B7A [NZ_AAJT01000066]	*BCDE*	17.9 b	49.9% [50.8%]	1
*Exiguobacterium* sp. AT1b [NZ_ABPF01000011]	*BCDE*	21.5	35.6% [48.4%]	0
*Geobacter uraniireducens* Rf4 [NC_009483]	*ABCDE*	17.9	51.4% [54.2%]	3
*Hahella chejuensis* KCTC 2396 [NC_007645]	*BCDE*	47.8	47.0% [53.9%]	4
*Mesorhizobium* sp. BNC1 Plasmid 3 [NC_008244]	*ABCDE*	47.5 c	61.5% [61.5%]	1
*Mycobacterium* abscessus ATCC 19977 [NC_010397]	*ABCDE*	19.6	59.0% [64.1%]	0
*Oceanobacter* sp. RED65 [NZ_AAQH01000003]	*BCDE*	22.0	40.7% [44.0%]	0
*Pseudoalteromonas haloplanktis* TAC125 chromosome II [NC_007482]	*ABCDE*	17.7	36.1% [39.4%]	0
*Pseudomonas fluorescens* PfO-1 [NC_007492]	*BCDE*	13.9	51.4% [60.5%]	2
*Roseobacter denitrificans* OCh 114 [NC_008209]	*ABCDE*	27.6	52.0% [59.0%]	2
*Salmonella enterica* serovar Saintpaul SARA23 [NZ_ABAM01000005]	*BCDE*	19.8	45.9% [52.4%]	0
*Shewanella pealeana* ATCC 700345 [NC_009901]	*BCDE*	22.5	39.3% [44.7%]	0
*Streptomyces avermitilis* MA-4680 [NC_003155]	*ABCDE*	29.0	62.8% [70.7%]	2
*Streptomyces lividans* 1326 [EF210454]	*ABCDE*	92.9	67.8% [70%]	2
*Vibrio cholerae* MZO-2 [NZ_AAWF01000002]	*BCDE*	15.0	45.0% [47.6%]	0
*Vibrio cholerae* MZO-3 [NZ_AAUU01000003 ]	*BCDE*	15.8	40.7% [47.0%]	0
*Vibrio fischeri* MJ11 chromosome II [NC_011186]	*BCDE*	17.9	39.5% [37.2%]	0

a The symbol ‘*A*’ indicates that the *dndA* gene is present in the reverse orientation with respect to the rest of the *dnd* gene cluster. The *dndBCDE* genes are invariably orientated in the same direction.

b The 17.9 kb represents the *leuX*-proximal part of the island within the unfinished *E. coli* B7A genome.

c The complete plasmid 3 was considered as a mobile element.

**Figure 2:**
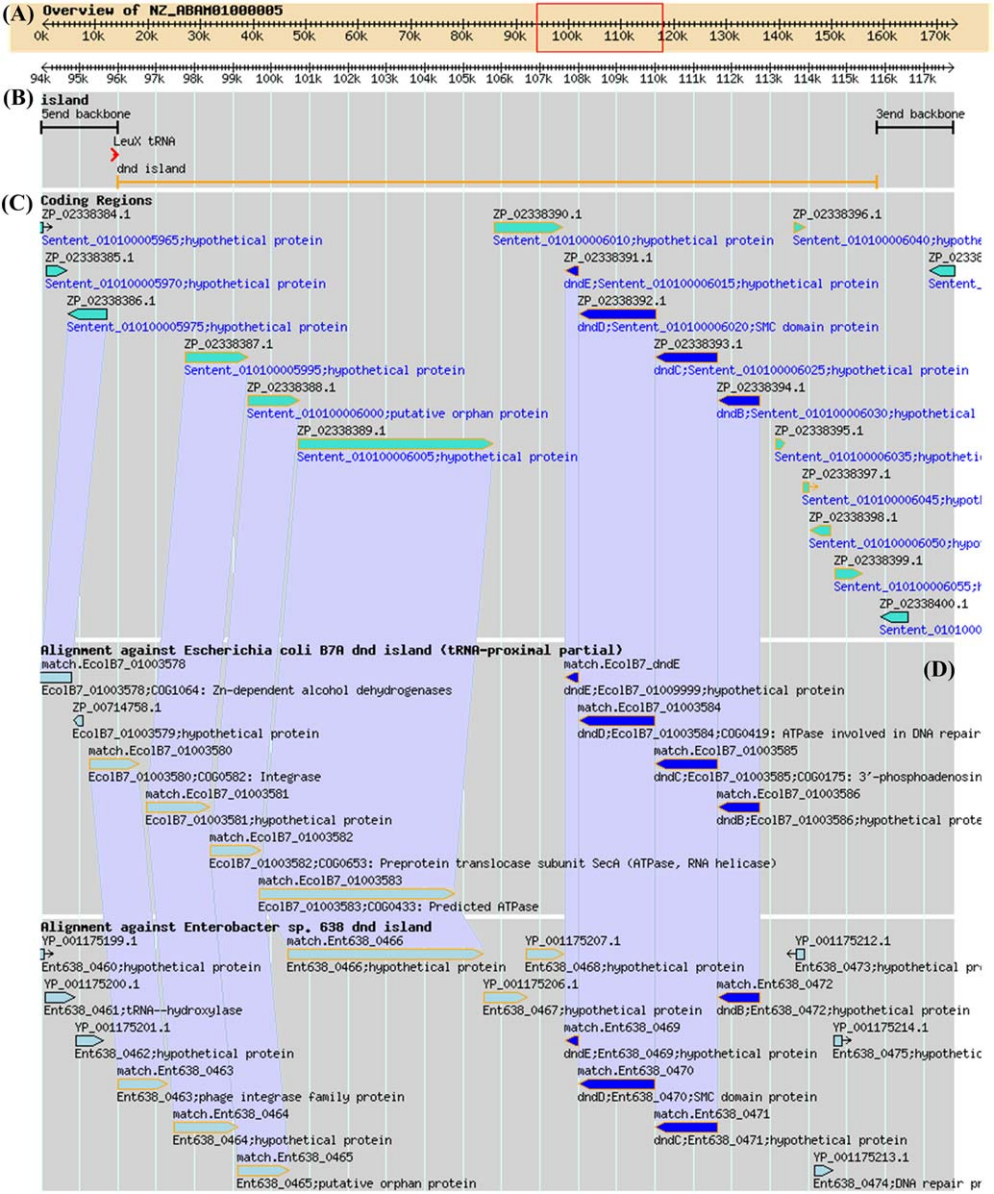
The *dnd* island in the *Salmonella enterica* serovar Saintpaul SARA23 genome that is currently being sequenced. (A) The top axis corresponds to the *dnd* island-bearing contig (NCBI Refseq accession no. NZ_ABAM01000005), while the lower axis represents a magnified view of the region shown in the red box. The symbol ‘k’ in the coordinates denotes kilobase pairs. (B) A schematic view of the lower axis (above) illustrating the location of the 19.7-kb *dnd* island (orange line), the *leuX* tRNA gene integration site (red arrow head), and the upstream/downstream flanking regions (black lines) that are conserved across 14 completely sequenced *Salmonella enterica* genomes. The ‘5end’ and ‘3end’ backbone labels refer to the 5′- and 3′-flanking backbone segments in relation to the orientation of the *leuX* tRNA gene, respectively. (C) SynView-facilated synteny mapping of the *dnd* islands and immediate flanking sequences from three species: *Salmonella enterica* serovar Saintpaul SARA23 (19.7-kb island) [topmost], *Escherichia coli* B7A (17.9-kb tRNA-proximal end of island) [middle] and *Enterobacter* sp. 638 (16.9-kb island) [lower most]. The *dnd* genes are highlighted in blue, while these and other island-harboured genes are marked by orange frames. Individual genes are hyperlinked to related information that can be accessed using GBrowse. Light-blue-shaded trapezoids link orthologous genes between the three species.

We have also incorporated the SynView tool [10] into *dnd*DB to facilitate larger scale synteny mapping so as to permit ready recognition of *dnd* island-borne orthologous genes. Figure 2 illustrates an example based on comparison of *dnd* islands from *Escherichia coli*, *Salmonella enterica* and *Enterobacter* sp. Such analyses will aid the identification of evolutionary links between members of this growing family of islands.

### Dnd proteins and conserved domains

Amino acid sequences of Dnd proteins from the diverse *dnd*-bearing hosts were multiply aligned with Muscle [7], visualised and edited with JalView [8]. The neighbor-joining phylogenetic tree of matching 16S rRNA sequences was constructed by using Muscle and JalView. A phylogenetic tree based on NCBI taxonomy IDs of host organisms was also generated by using iTOL [11]. *dnd*DB also contains a list of conserved domains and consensus sequences identified in Dnd proteins that have been previously deposited in the protein family database Pfam, the Conserved Domain Database (CCD), and/or the biological macromolecule 3-D structures database PDB [12]. In addition, hundreds of other proteins exhibiting lower levels of similarity to Dnd proteins with Blastp E-values of less than E −4 were extracted from the NCBI nr database and stored in *dnd*DB to allow for rapid identification of more distantly related potential homologues or proteins performing related functions.

**Figure 3:**
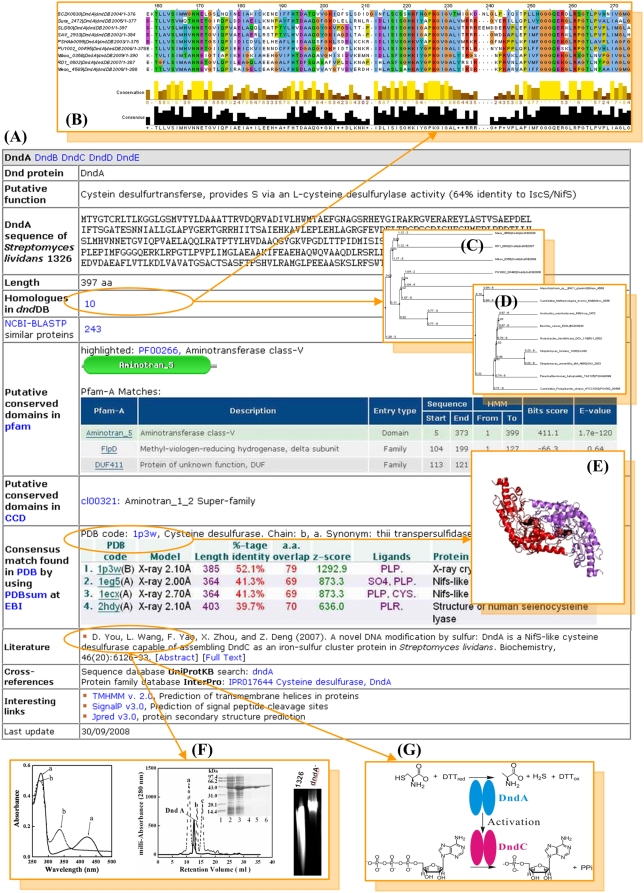
Organization of Dnd proteins and conserved domains in *dnd*DB. (A) DndA protein data that have been used to predict its putative biological function as a likely cysteine desulfur-transferase in *Streptomyces lividans*. (B) Multiple amino acid sequence alignment of DndA proteins highlighted the conserved domain in Pfam (accession no. PF00266). (C) and (D) Phylogenetic trees drawn on basis of DndA amino acid sequences and 16S rDNA sequences of the host organisms, respectively. (E) A 3-D structural image corresponding to a DndA-related protein (PDB ID: 1p3w). (G) Sample experimental data demonstrating that DndA provides sulphur via its L-cysteine desulfurylase activity [13]. (F) Inferred biochemical reaction, in which DndA is predicted to catalyze the assembly of DndC as an iron–sulfur cluster protein [13].

We have used *dnd*DB and associated experimentation to analyse the DndA, DndB, DndC, DndD and DndE proteins of *S. lividans* and have used these data to predict their putative biological functions, thus shedding light on the novel DNA phosphorothioation biochemical pathway. The DndA protein is a likely cysteine desulfur-transferase that is proposed to provide sulphur via its L-cysteine desulfurylase activity (see Figure 3 for an outline of relevant data) [13]. DndB is a predicted Fe-S cluster binding protein, which we hypothesize affects modification specificity through its action as a transcriptional regulator. Similarly, DndC is proposed to contain a [4Fe-4S] cluster and has predicted ATP pyrophosphatase activity, features paralleling those of IscS and ThiI [14] which are involved in tRNA sulfur modification in *Escherichia coli*. DndD is a putative ATPase with DNA nicking activity which may couple ATP hydrolysis to DndE, a putative sulphur-transferase. However, much more detailed analyses and experimentation will be necessary to finalize the precise nature of the *dnd* biochemical pathway.

### Search tools

The *dnd*DB web server offers several search tools with varied options. Through the ‘Search’ page, users can retrieve Dnd phenotype, gene or protein homologues from *dnd*DB by organism name. Via the ‘Blast vs *dnd*DB’ page, users are able to blast a query sequence against *dnd*DB to find homologous matches with WU-BLAST 2.0 [Gish, W., personal communication]. Finally, the ‘tBlastn for Dnd’ page, utilizes a NCBI tBlastn-based tool that we developed to predict potential Dnd proteins in user-supplied nucleotide sequences.

As future developments, we will shortly be uploading a large set of sequences which exhibit homology to isolated *dnd* genes, as apposed to *dnd* clusters only, and a further set corresponding to homologues of the full complement of non-*dnd* genes borne on *dnd* islands. We will continue to identify additional syntenic clusters, isolated *dnd*-like genes and other *dnd* island gene homologues as gene, genome and metagenome databases expand, and anticipate eventually providing a pipeline for ready automated discovery, annotation and analyses of *dnd* genes, clusters and associated genomic islands.

We envisage an evolving resource that seeks to effectively combine and interlink the genetics, biochemistry and functional aspects of *dnd* systems and their associated genomic islands. Such a unified resource will facilitate efficient investigation of a wide range of aspects relating to *dnd* DNA modification processes and other island-encoded functions in diverse host organisms. We also believe that the lessons learnt from ongoing dissection of the *dnd* system will provide clues to resolve mysteries relating to weakly similar genes, proteins and biochemical reactions, and in due course give rise to novel biotechnological and/or clinical applications; thus we expect that *dnd*DB will prove to be of interest to a broad community of researchers.

## Materials and Methods

The *dnd*DB database runs on a Linux platform (Fedora core 5) with the Apache web-server (version 2.2.0), MySQL server (v 5.0.22), PHP (v 5.1.4), Perl (v 5.8.8) and Bioperl (v 2.1) [15]. In addition, the following freely available components were employed: NCBI Blast 2.2.9 [16], WU-BLAST 2.0 [Gish, W., personal communication], Muscle 3.7 [7], GBrowse 1.69 [9] and JalView 2.4 [8]. *dnd*DB version 1.0 is freely available for research activities and non-commercial use at http://mml.sjtu.edu.cn/dndDB/. The Java platform (http://www.java.com/) is required for web browser-based visualisation of Muscle-generated phylogenetic trees using JalView.

The current version of *dnd*DB includes the following information. (i) List of 24 bacterial species exhibiting the Dnd phenotype and associated publications; (ii) Details of *dnd* gene clusters from 25 species of Eubacteria and Archaea that were identified based on both sequence similarity and gene order (synteny) by employing Blastp searches against complete and partially sequenced genomes available at the NCBI server; (iii) Details of laterally acquired genomic islands harbouring *dnd* genes that were predicted using the GBrowse viewer (Generic Genome Browser) [9], MobilomeFINDER server [17], Z Curve database online utility [18] and/or interactive Artemis Comparison Tool (WebACT) [19]. (iv) Archive of Dnd proteins and other potentially related proteins showing BLAST-based similarity, and corresponding conserved domains identified in the protein family database Pfam [20] and the Conserved Domain Database (CCD) [21].

*dnd*DB currently contains details of over 114 *dnd* genes and their cognate proteins from the Eubacterial and Archaeal kingdoms, and is expected to grow quickly with the rapid development of genome sequencing projects and the ongoing refinement of strategies to identify distantly related gene clusters, orphan *dnd* genes, and functionally or biochemically related proteins. As more information about the Dnd system becomes available, the database will be expanded and improved accordingly.

In addition, brief descriptions of ongoing research into the *dnd* system by our group and collaborators are also incorporated into *dnd*DB to foster dialogue and participation by the wider research community. These include work on a putative Dnd-dependent restriction-modification system, the precise nature of the DNA modification itself, the core sequence motif that targets the site-specific modification in *S. lividans* [22], and the increasingly well characterized novel biochemical pathway that mediates this unique biological process. Future contributions from other researchers will be sought via *dnd*DB.
